# Acetic acid: a cheap but chief metabolic regulator for abiotic stress tolerance in plants

**DOI:** 10.1007/s44154-024-00167-9

**Published:** 2024-07-29

**Authors:** Md. Mezanur Rahman, Sanjida Sultana Keya, Abira Sahu, Aarti Gupta, Anuradha Dhingra, Lam-Son Phan Tran, Mohammad Golam Mostofa

**Affiliations:** 1 Department of Plant and Soil Science, Institute of Genomics for Crop Abiotic Stress Tolerance, Texas Tech University, Lubbock, TX 79409 USA; 2 Department of Energy Plant Research Laboratory, Michigan State University, East Lansing, MI 48824 USA; 3 Department of Biology, Washington University in St. Louis, St. Louis, USA; 4 Department of Biochemistry and Molecular Biology, Michigan State University, East Lansing, MI 48824 USA

**Keywords:** Acetic acid, Ecofriendly, Metabolic regulation, Signaling molecules, Stress adaptation, Plant metabolites

## Abstract

As sessile organisms, plants constantly face a variety of abiotic stresses, such as drought, salinity, and metal/metalloid toxicity, all of which possess significant threats to plant growth and yield potential. Improving plant resilience to such abiotic stresses bears paramount importance in practicing sustainable agriculture worldwide. Acetic acid/acetate has been recognized as an important metabolite with multifaceted roles in regulating plant adaptation to diverse abiotic stresses. Recent studies have elucidated that acetic acid can potentiate plants’ inherent mechanisms to withstand the adverse effects of abiotic stresses through the regulation of lipid metabolism, hormone signaling, epigenetic changes, and physiological defense mechanisms. Numerous studies also underpin the potential use of acetic acid in boosting crop production under unfavorable environmental conditions. This review provides a comprehensive update on the understanding of how acetic acid regulates plant photosynthesis, acts as an antitranspirant, detoxifies reactive oxygen species to alleviate oxidative stress, interacts with phytohormones to regulate physiological processes, and improves soil fertility and microbial diversity, with a specific focus on drought, salinity, and metal toxicity. We also highlight the eco-friendly and economic potential of acetic acid that may attract farmers from developing countries to harness the benefits of acetic acid application for boosting abiotic stress resistance in crops. Given that acetic acid is a widely accessible, inexpensive, and eco-friendly compound, the revelation of acetic acid-mediated regulatory pathways and its crosstalk with other signaling molecules will have significant importance in developing a sustainable strategy for mitigating abiotic stresses in crops.

## Introduction

Acetic acid (CH_3_COOH, acetate) in vinegar is a well-recognized ancient preservative (Doran 1928), which is manufactured through a robust process of anaerobic fermentation and diverse industrial chemical methods (Deshmukh and Manyar 2021). Uncovering the latent strength of acetic acid in enhancing plant ability to withstand abiotic stressors has taken nearly a decade of investigation. Kim et al. (2017) unveiled a novel yet straightforward role of acetic acid in fortifying drought tolerance of several plant species by exposing them to diluted acetic acid (0, 10, 20, 30, and 50 mM). Moving forward, a lot of recent studies provided experimental evidence that application of exogenous acetic acid mitigated negative consequences of various abiotic stresses, including drought (Kim et al. 2017; Rasheed et al. 2018; Utsumi et al. 2019; Rahman et al. 2021), salinity (Rahman et al. 2019), and heavy metal toxicity (Hossain et al. 2020; Wang et al. 2023). Acetic acid-mediated augmentation of plant resistance to abiotic stresses includes the improvement of specific physiological and biochemical functions that regulate growth and development of both roots and shoots, net photosynthetic rate, water-use efficiency, stomatal conductance, chlorophyll biosynthesis, leaf water status, nutrient-use efficiency, and activation of antioxidant defense (Rahman et al. 2019, 2021; Utsumi et al. 2019; Hossain et al. 2020). Acetic acid plays a key role in upregulating the expression of the jasmonic acid (JA) biosynthetic gene *ALLENE OXIDE CYCLASE 3* (*AOC3*), and the subsequent improvement of JA level plays a critical epigenetic role in promoting drought resistance in Arabidopsis plants (Kim et al. 2017). However, they did not observe the substantial effect of acetic acid in changing abscisic acid (ABA) level under drought conditions. Whereas, in cassava (*Manihot esculenta*) and *Carex rigescens* plants, acetic acid was found to regulate the level of ABA to control stomatal movement and various lipids to fortify the membrane’s robustness and integrity (Utsumi et al. 2019; Hu et al. 2021). The interplay between acetic acid and phytohormones under salinity stress seems intricate and depends on the types of plant species. For instance, acetic acid increased the level of salicylic acid (SA) in grapevines (*Vitis vinifera*), while it boosted indole-3-acetic acid (IAA) and gibberellic acid levels but reduced the level of ABA in wheat (*Triticum aestivum*) (Lv et al. 2021; Khan et al. 2022a). Indeed, the effect of acetic acid on hormonal biosynthesis and signaling is a complex and species-specific process, underlining the importance of taking environmental factors and plant species into account to fully comprehend its effects. Albeit the role of acetic acid in modulating rhizosphere soil compositions and, thus, soil microbial diversity is only emerging (Kong et al. 2022), it demands extensive investigation under a variety of stress and soil conditions to fully understand its potential.

The paramount crisis facing the world today is how to nurture the anticipated 9.8 billion population by 2050 in the context of climate change and shortage of arable land (United Nations 2017). These circumstances necessitate an immediate and decisive advanced crop breeding technologies to attain maximum crop yields for sustainable agricultural output (Tripathi et al. 2019). Regrettably, the advancement of sustainable agricultural development is severely perturbed by a multitude of climate change-induced abiotic stresses, encompassing soil salinization, dire water scarcity, temperature fluctuations, and toxic heavy metal contamination of agricultural lands (Zhang et al. 2022). These abiotic stress factors constrain the worldwide use of arable agricultural lands, resulting in a formidable decline in crop productivity. For instance, soil salinization engulfs more than a billion hectares of the world’s arid and semi-arid regions, leading to a catastrophic financial loss of over $27 billion in crop value every year (FAO 2021; Rahman et al. 2021; Sahab et al. 2021; Hassani et al. 2021). Over the past two decades, drought has affected 1.9 billion hectares of land, jeopardizing the lives of nearly 1.5 billion individuals, and incurring losses of nearly US$ 124 billion globally (Erian et al. 2021). Due to rampant industrialization and impromptu management practices, the pollution of soil by heavy metals and metalloids is becoming a looming global pandemic, unless decisive measures are taken to curb its expansion (Oladoye et al. 2021).

Plants are subjected to numerous abiotic stresses, either alone or in combination, leading to severe morphological, physiological, and metabolic anomalies, including diminished water uptake, compromised photosynthetic efficiency, hindered nutrient uptake and distribution, cell shrinkage, loss of cell turgor, and overproduction of reactive oxygen species (ROS) (Zhang et al. 2021). To thrive under such conditions, plants undergo a plethora of morpho-physiological adaptations, which include but are not limited to, modifications in root system architecture, stomatal closure, arrest of vegetative growth, reinforcement of antioxidant defenses, and osmoprotectant accumulation (Zhang et al. 2021; Omae and Tsuda 2022; Xu et al. 2022). Although these physiological adaptations serve as survival mechanisms, plants can still suffer from deleterious effects of abiotic stresses that can significantly impact the yield output.

An array of strategies, such as the identification and isolation of novel genes, selective breeding, and cutting-edge biotechnological methodologies can be employed to engineer plants with enhanced resilience to adverse environmental conditions. Alternatively, the application of exogenous chemicals has garnered significant attention as a potential strategy to enhance plant resilience to environmental stresses, including drought, salinity, and heavy metal toxicity, due to its immediate impacts (Savvides et al. 2016; Cao et al. 2017). In this context, acetic acid has gained significant attention as a potent player in addressing abiotic stress problems in agricultural systems, as it is inexpensive, readily available, and ecofriendly at low concentrations. In this review, we explore the current comprehended information on the metabolic processes of acetic acid in plants and the technologies employed for the large-scale production of acetic acid. We succinctly highlight the economically and environmentally friendly potential aspect of acetic acid in practicing abiotic stress mitigation strategies in crops. We also delve into the recent key findings on the crucial involvement of acetic acid in regulating the morphological and physiological processes, metabolic pathways, and hormonal levels that are essential for elevating plant responses to abiotic stresses.

## Acetate metabolism in plants and it’s commercial production

**Fig. 1:**
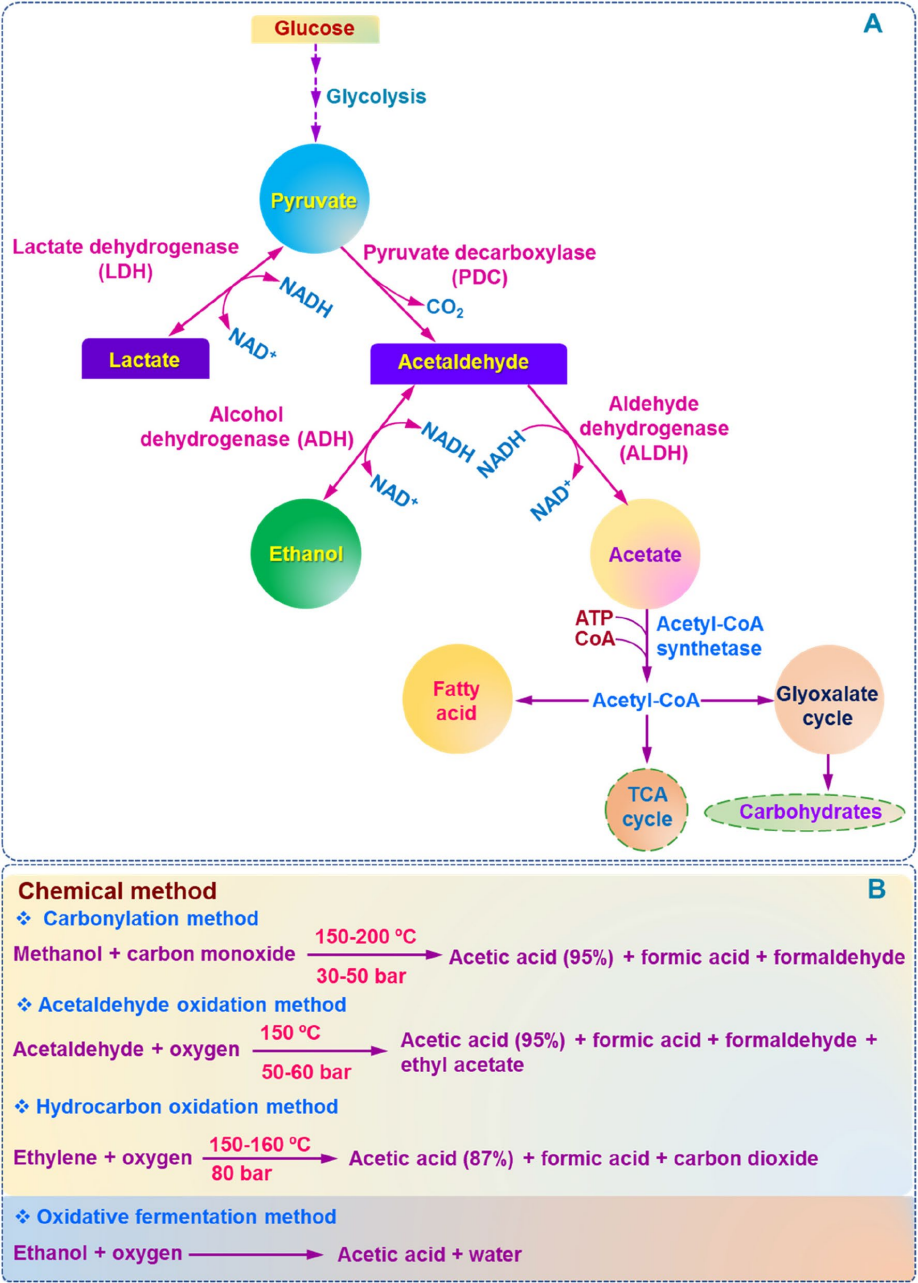
Pathways linked to acetate metabolism in plants. **A** The process of glycolysis converts glucose into pyruvate. Under anaerobic conditions, pyruvate is converted to lactate by the lactate dehydrogenase (LDH) enzyme. Simultaneously, pyruvate can undergo a two-step process to transform into acetate. The first step is the conversion of pyruvate to acetaldehyde by the pyruvate decarboxylase (PDC) enzyme, followed by the conversion of acetaldehyde to acetate by the aldehyde dehydrogenase (ALDH) enzyme. Alternatively, acetaldehyde can be converted to ethanol by the alcohol dehydrogenase (ADH) enzyme. However, the acetate can be converted to acetyl-CoA by the acetyl-CoA synthetase. Acetyl-CoA formed in the plastid by this reaction can be utilized for fatty acid biosynthesis. The carbon may also be converted to carbohydrates through the glyoxylate shunt. **B** In commercial production, acetic acid can be produced through two primary methods: chemical and fermentation. Chemical methods involve various techniques such as carbonylation, acetaldehyde oxidation, and hydrocarbon oxidation. On the other hand, the oxidative fermentation technique utilizes ethanol as a precursor to manufacture acetic acid

The biosynthesis of acetate  in plants commences with pyruvate, which is produced from glucose through glycolysis. In anaerobic conditions, including submergence and waterlogging, plants employ the enzyme lactate dehydrogenase (LDH) to convert pyruvate into lactate using NADH (nicotinamide adenine dinucleotide) as a cofactor (Fig. 1A) (Tadege et al. 1999). Simultaneously, pyruvate can be decarboxylated into acetaldehyde via pyruvate decarboxylase. Subsequently, acetaldehyde transforms into ethanol with the assistance of alcohol dehydrogenase (ADH), resulting in the oxidation of NADH (Fig. 1A). It is important to highlight that acetaldehyde can also be converted into acetate, facilitated by the aldehyde dehydrogenase (ALDH) (Fig. 1A) (Tadege et al. 1999; Rasheed et al. 2018). The acetate molecule has the potential to be transformed into acetyl-CoA by acetyl-CoA synthetase. Once acetyl-CoA is produced within the plastid, it can be effectively employed in the biosynthesis of fatty acids. Additionally, the carbon molecule from acetate can be channeled toward the synthesis of carbohydrates via the glyoxylate shunt (Fig. 1A). Acetic acid is predominantly utilized as a vital raw material or an intermediate component in the synthesis of a range of crucial chemicals, including vinyl acetate monomer, polyethylene terephthalate (PET), ethyl acetate, and cellulose acetate (Medrano-García et al. 2019). The growing demand from these chemical industries has heightened the need for acetic acid production, as evidenced by the current production rate of 16.7 million metric tons, valued at USD 20.6 billion, which is expected to soar to USD 31.9 billion by 2030 (available at https://www.reportlinker.com/p06360868/?utm_source=GNW). To fulfill the current industrial demand, both chemical and fermentative production methods are widely utilized. The three main chemical manufacturing processes include the Cavita process, which entails the carbonylation of methanol, as well as oxidizing aldehydes, and hydrocarbon oxidation (Fig. 1B). The perpetual demand for acetic acid necessitates the advancement of eco-friendly production techniques. Fermentative production of acetic acid at a small scale has garnered significant attention in recent decades; however, a commercialized strategy is yet to be established (Medrano-García et al. 2019).

## Acetic acid is an eco-friendly and cost-effective biostimulant

The issue of whether acetic acid is harmful to the environment is far more complex than a straightforward answer of ‘yes’ or ‘no’. The determination of the environmental impact of acetic acid hinges on a multitude of considerations, including the origin of the raw materials used and the extent of its dilution. For the majority of plant science experiments that are focused on abiotic stress tolerance, researchers have employed very low concentrations of acetic acid, within a range of 0.0585% (10 mM) to 0.580% (100 mM). Besides having roles in augmenting plant tolerance to abiotic stresses, acetic acid has been identified as a promising alternative to glyphosate for weed control at the concentration ranges from 10–20% (Garcia and Youngblood 2017; Pujisiswanto et al. 2013; Domenghini 2020). Indeed, higher concentrations of acetic acid result in more effective weed control (available at https://attra.ncat.org/publication/sustainable-weed-management-for-small-and-medium-sized-farms/). Notably, acetic acid with a concentration of 8% or less falls under the exemption from registration as a pesticide by the Environmental Protection Agency (EPA) under the provisions of the Federal Insecticide, Fungicide, and Rodenticide Act (FIFRA) 25(b) as an inert ingredient of Minimum Risk Pesticide (available at https://extension.umd.edu/resource/vinegar-alternative-glyphosate). This signifies that the concentrations utilized by plant researchers for abiotic stresses are much safer for the soil environment as well as for aquatic plants and animals. Fundamentally, acetic acid dissociates into the acetate anion and hydrogen proton in water, with the acetate anion exhibiting high biodegradability, as 99% degradation is observed after just 7 days under anaerobic conditions in the presence of activated sludge (American Chemistry Council, 2001). Nevertheless, further research is necessary to attain more precise information about the optimal application rate and timing, long-term effects on soil health, and crop-specific responses. It is important to emphasize, based on existing literature, that the optimal concentration of acetic acid, proven to provide maximum protection to plants against the adversities of drought and salinity, was around 20 mM. Thus, it can be undeniably proclaimed that acetic acid at its optimal concentration of 20 mM, embodies a remarkably economical choice, costing a mere $0.4 per kiloliter, as reported by ICIS Chemical News in 2020 and valued at a remarkably low $330 per ton (ICIS Chemical News 2020; Allen and Allen 2020). In edible plants, acetic acid is known to be used as preservative to protect antioxidant properties (Alm 1952). For instance, acetic acid application has been found to significantly reduce the oxidation of ascorbic acid in parsley (*Petroselinum crispum*), spinach (*Spinacia oleracea*), cauliflower (*Brassica oleracea*), and tomato (*Solanum lycopersicum*) by 80–50%, and in orange (*Citrus sinensis*) and onion (*Allium cepa*) by 90–100% (Alm 1952). It would be interesting to examine whether acetic acid provides any protections to other nutritional properties in edible, nonedible, and bioenergy crops. Likewise, determining whether acetic acid imparts any lingering effects on nutraceutical attributes in edible plants would help determine the potential implications of acetic acid in human health. In essence, considering its environmentally friendly nature, acetic acid utilization for boosting plant abiotic stress tolerance might contribute to enhance crop yield without encountering any adverse impacts on crop quality and arable lands.

## The role of acetic acid in mitigating abiotic stresses in plants

The function of acetic acid as a natural safeguarding agent against environmental stressors has drawn significant attention. It actively participates in diverse physiological processes, including growth enhancement, root proliferation, seed sprouting, photosynthesis, antioxidant defense, and osmoregulation that play important roles in plant survival under stressful conditions. The intricate mechanisms of acetic acid-mediated abiotic stress (drought, salinity, metal toxicity, and cold) tolerance  are succinctly summarized below.

### The role of acetic acid in plants under drought conditions

**Fig. 2:**
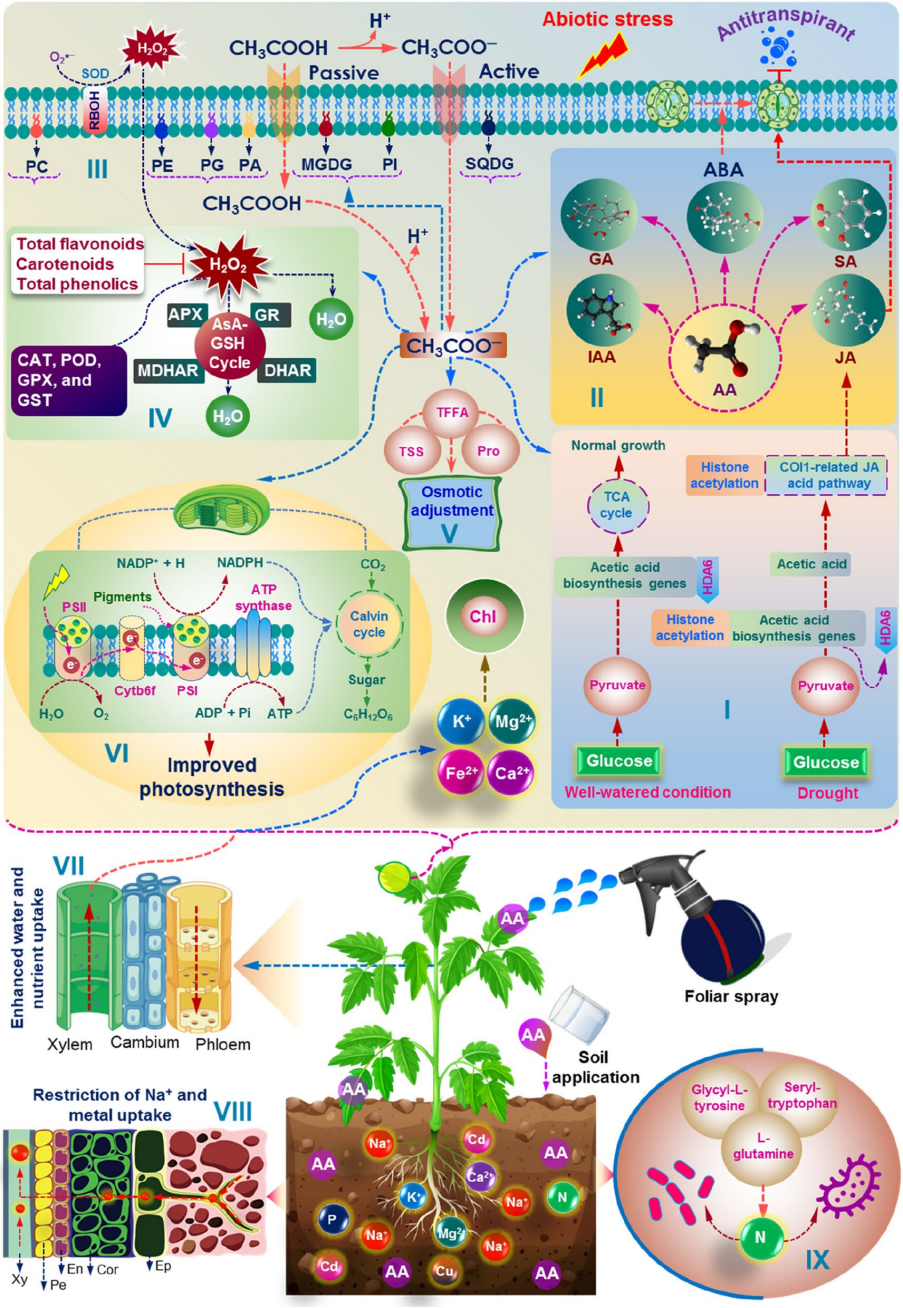
Putative role of acetic acid in mitigating abiotic stresses in plants. Supplementing plants with exogenous acetic acid in the form of a foliar spray or soil amendment can increase cellular acetic acid levels, leading to improved plant resilience to various abiotic stressors. This is achieved by several mechanisms, including (**i**) modulating epigenetic mechanisms in plants: under well-watered conditions, histone deacetylase 6 (HDA6) suppresses the expression of acetic acid biosynthesis genes *PDC1* and *ALDH2B7* by restricting histone H4 acetylation enrichment at their transcribed gene body regions. In contrast, under drought conditions, HDA6 dissociates from *PDC1* and *ALDH2B7*, leading to a surge in H4 acetylation enrichment and transcriptional activation of these two genes. This results in increased acetate accumulation, which then stimulates the JA signaling pathway, enhancing the plant’s ability to withstand drought stress; (**ii**) regulating phytohormone levels: exogenous acetic acid supplementation can regulate the levels of various phytohormones, including GA, IAA, SA, JA, and ABA. ABA and JA play a synergistic role in partially closing stomata to reduce water loss and serve as a protective antitranspirant mechanism; (**iii**) strengthening membrane stability and integrity: increased accumulation of lipids such as phosphatidylcholine, phosphatidylethanolamine, phosphatidylglycerol, monogalactosyl diacylglycerol, galactosyl diacylglycerol, and sulfoquinovosyldiacylglycerl can improve membrane stability and integrity; **(iv)** increasing detoxification of reactive oxygen species (ROS): elevated antioxidant enzyme activities and increased levels of non-enzymatic antioxidants helping to detoxify ROS; **(v)** accumulating osmoprotectants: exogenous acetic acid supplementation can increase the accumulation of osmoprotectants, like total soluble sugars, total free amino acids and proline, which help maintain water balance in plants; (**vi**) increasing photosynthetic activity: increasing activity of photosystem II can enhance photosynthetic activity; (**vii** and **viii**) restricting the accumulation of toxic ions: increased acetic acid accumulation can restrict the uptake of toxic ions such as Na^+^ and heavy metals while elevating the levels of essential minerals that partially contribute to chlorophyll synthesis and photosynthesis; (**ix**) improving root function: increased acetic acid accumulation in roots can help in increasing soil metabolites that increase nitrogen availability, which is linked to the abundance of microorganisms like *Azotobacter* and *Pseudomonas*. Abbreviations: ABA (abscisic acid), AA (acetic acid), ADP (adenosine diphosphate), APX (ascorbate peroxidase), ATP (adenosine triphosphate), AsA (ascorbic acid), CH_3_COOH (acetic acid), Ca^2+^ (calcium ion), CAT (catalase), Cd (cadmium), Chl (chlorophyll), CO_2_ (carbon dioxide), Cor (cortex), Cu (copper), Cytb6f (cytochrome b6f), DHAR (dehydroascorbate reductase), En (endodermis), Ep (epidermis), Fe^2^^+^ (iron) GA (gibberellic acid), GPX (glutathione peroxidase), GR (glutathione reductase), GSH (glutathione), GST (glutathione *S* transferase), H^+^ (hydrogen ion), H (hydrogen), H_2_O (water), H_2_O_2_ (hydrogen peroxide), IAA (auxin), JA (jasmonic acid), K^+^ (potassium ion), MDHAR (mono-dehydroascorbate reductase), Mg^2+^ (magnesium ion), MGDG (monogalactosyl diacylglycerol), N (nitrogen), Na^+^ (sodium ion), NADP^+^ (oxidized nicotinamide adenine dinucleotide phosphate), NADPH (reduced nicotinamide adenine dinucleotide phosphate), O_2_ (oxygen), O_2_•^–^ (superoxide ion), P (phosphorus), PA (phosphatidic acid), PC (phosphatidyl-cholines), PE (phosphatidylethanolamine), Pe (pericycle), PG (phosphatidylglycerol), PI (phosphatidylinositol), Pi (inorganic phosphate), PSI (photosystem I), PSII (photosystem II), POD (peroxidase), Pro (proline), RBOH (respiratory burst oxidase homolog), SA (salicylic acid), SOD (superoxide dismutase), SQDG (sulfoquinovosyldiacylglycerol), TFFA (total free amino acids), TSS (total soluble sugars), Xy (xylem)

An abundance of research has demonstrated that the application of acetic acid plays a crucial role in promoting and maintaining the growth of plants under drought stress. Kim et al. (2017) made a seminal contribution by presenting the initial genetic and epigenetic proof of acetic acid’s pivotal role in drought tolerance using wild type (WT) and *histone deacetylase6* (*hda6*) mutant of Arabidopsis. This study revealed that during drought stress, the synthesis of acetic acid was triggered through an increased expression of the genes encoding *pyruvate decarboxylase* (*PDC1*) and *aldehyde dehydrogenase* (*ALDH2B7*) in the WT plants (Fig. 2) (Table 1). This crucial role of acetic acid in drought resistance was further validated by Rasheed et al. (2018), where the upregulation of genes associated with acetic acid biosynthesis pathways, namely *PDC1* or *ALDH2B7*, using an outer membrane *tryptophan-rich sensory protein* (*TSPO*) promoter, substantially strengthened the drought stress tolerance of transgenic Arabidopsis plants (Table 1). Interestingly, the direct application of exogenous acetic acid did not stimulate the JA-responsive genes. Instead, it was only through subsequent exposure to drought stress that these genes were activated, implying that acetic acid acts as a priming mechanism for the induction of JA-responsive genes in Arabidopsis (Kim et al. 2017).

**Table 1:** The potential roles of acetic acid in improving adaptivity in plants exposed to various forms of abiotic stresses

Abiotic stress	Plant species	Stress treatment and duration of stress exposure	Mode of application and acetic acid concentration(s)	Acetic acid-induced protective mechanisms	References
Drought	*Arabidopsis thaliana*	Water withheld, 14 days	Pretreatment to soil (0, 1, 10, 20, 30, and 50 mM)	Priming of JA signaling pathway-related genes, which helps in drought tolerance during drought exposure; epigenetic regulation	Kim et al. 2017
Drought	*Arabidopsis thaliana*	Water withheld, 11 days	Acetic acid-rich transgenic plants	Increased the expression of genes, *PDC1* and *ALDH2B7*, related to acetic acid biosynthesis	Rasheed et al. 2018
Drought	*Manihot esculenta*	Water withheld, 14 days	Pretreatment to soil (1 and 10 mM)	Increased the expression of ABA signaling pathway-related genes (*OST1* and *PP2C*), and subsequently enhanced ABA content; reduced stomatal conductance and transpiration rate to maintain RWC; enhanced the expression of drought response and tolerance-linked genes like *TSPO* and *HSPs*	Utsumi et al. 2019
Drought	*Gossypium hirsutum*	Water withheld, 9 days	Pretreatment to soil (2, 4, 6, and 8 mM)	Upregulated the expression of genes related to ABA biosynthesis (*NCED2*, *NCED3*, and *NCED9*) and JA biosynthesis (*GhAOS6*, *GhLOX3*, and *GhOPR11*); decreased stomatal conductance and transpiration rate	Li et al. 2021
Drought	*Begonia* × *hybrida*	Manually deploying transparent polythene sheet considering weather forecast, 28 days	Pretreatment to soil (0, 10, 20, and 38 mM)	Increased shelf life of begonia by around 9 days by reducing the rate of tray evapotranspiration	Allen and Allen 2021
Drought	*Oryza sativa*	Water withheld, 4 days	Pretreatment to soil (30 mM)	Activates root-to-shoot JA signaling; increased ABA accumulation in the root; decreased transpiration rate	Ogawa et al. 2021
Drought	*Glycine max*	Water withheld, 11 days	Foliar spray (20 mM)	Increased antioxidant enzyme activities, including SOD, CAT, APX, GPX, and GST; reduced the levels of superoxide, hydrogen peroxide, MDA, and electrolyte leakage; increased the levels of RWC, soluble sugars, and free amino acids; increased essential mineral acquisition in roots, stems, and leaves; enhanced Chl contents and photosynthetic level	Rahman et al. 2021
Drought	*Salix myrtillacea*	Maintained soil relative water capacity at 40% for 20 days	Pretreatment to soil (100 mM)	Increased antioxidant enzyme activities, including SOD, POD, and CAT; reduced the levels of superoxide, hydrogen peroxide, and MDA; reduced ABA content in both roots and leaves, while increased SA and JA level in leaves; increased the levels of soil metabolites, including glycyl-L-tyrosine, L-glutamine, and seryl-tryptophan; reduced the levels of phenylpropane metabolites, such as fraxetin and sinapyl aldehyde; increased nitrogen availability; enhanced the abundance of *Azotobacter* and *Pseudomonas*	Kong et al. 2022
Drought	*Malus domestica*	Water withheld, 15 days	Pretreatment to soil (10 mM)	Increased antioxidant enzyme activities, including SOD, POD, and CAT; reduced the levels of hydrogen peroxide, MDA, and electrolyte leakage; increased Chl levels and the rate of photosynthesis and transpiration; increased the levels of ABA, JA, and MeJA	Sun et al. 2022
Drought	*Cunninghamia lanceolata*	Maintained soil volumetric water content at 20% for 30 days	Pretreatment to soil (200 mM)	Increased enzymatic antioxidant activities, like SOD, as well as the levels of non-enzymatic antioxidants, such as total phenol and flavonoids; reduced the levels of hydrogen peroxide, MDA, and electrolyte leakage; increased chlorophyll pigments level and the activity of photosystem II reaction center, and thereby photosynthetic rate; increased the level of soluble sugars and plant water status	Li et al. 2022
Drought	*Pteris vittate*	Water withheld, 32 days	Pretreatment to soil (10 and 20 mM)	Increased RWC in shoots	Wang et al. 2023
Salinity	*Vigna radiata*	80- and 160-mM seawater, 14 days	Foliar application (20 mM)	Increased enzymatic antioxidant activities, like CAT, as well as the levels of non-enzymatic antioxidants, such as total phenolics and total flavonoids; reduced the levels of hydrogen peroxide, MDA, and electrolyte leakage; increased Chl levels and the rate of photosynthesis; increased the levels of proline, total free amino acids, and soluble sugars; restricted the accumulation of toxic Na^+^, while enhanced other minerals uptake; increased leaf succulence	Rahman et al. 2019
Salinity	*Lens culinaris*	100 mM NaCl, 15 days	Added to the nutrient solution (5, 10, and 20 mM)	Increased enzymatic antioxidant activities, like CAT, as well as the levels of non-enzymatic antioxidants, such as AsA; reduced the levels of MDA and electrolyte leakage; restricted the accumulation of toxic Na^+^, while enhanced the uptake of K^+^; increased Chl levels; enhanced leaf relative water content	Hossain et al. 2019
Salinity	*Lolium perenne*	250 mM NaCl, 25 days	Pretreatment to soil (20 mM)	Increased enzymatic antioxidant activities, like SOD and CAT; reduced the levels of superoxide, hydrogen peroxide, and electrolyte leakage; restricted the accumulation of toxic Na^+^, while enhancing the uptake of K^+^; enhanced the expression of genes related to JA, IAA, and CK biosynthesis, as well as downregulated the ABA biosynthesis genes; increased the levels of JA, IAA, CK, and reduced the content of ABA; increased Chl levels; increased photochemical efficiency (Fv/Fm) of the photosystem II; enhanced endogenous acetic acid level	Zhang et al. 2021
Salinity	*Carex rigescens*	300 mM NaCl, 15 days	Pretreatment to soil (30 mM)	Enhanced the accumulation of lipids such as phosphatidylcholine, phosphatidylethanolamine, phosphatidylglycerol, monogalactosyl diacylglycerol, galactosyl diacylglycerol, and sulfoquinovosyldiacylglycerol; increased the accumulation of lipid signaling molecules and their precursors, like phosphatidic acid, phosphatidylcholine and phosphatidylinositol; reduced the accumulation of toxic lipid intermediates such as free fatty acids; reduced the levels of MDA and electrolyte leakages	Hu et al. 2021
Salinity	*Triticum aestivum*	70- and 140-mM NaCl, throughout the growing cycle	Foliar application (10 and 20 mM)	Increased antioxidant enzyme activities, including SOD, POD, APX, and CAT; reduced the levels of hydrogen peroxide and MDA; increased Chl levels and the rate of photosynthesis; enhanced the levels of total soluble sugars, free amino acids, and leaf RWC; increased the levels of auxin and gibberellic acid, while reduced the content of ABA; restricted the accumulation of toxic Na^+^, while enhanced other minerals uptake, like K^+^, Ca^2+^, Mg^2+^ and nitrogen; improvement of yield-related traits	Khan et al. 2022a
Salinity	*Triticum aestivum*	60- and 120-mM NaCl, throughout the growing cycle	Foliar application (15 and 30 mM)	Increased antioxidant enzyme activities, including CAT and POD; reduced the levels of hydrogen peroxide, and electrolyte leakage; enhanced the levels of total soluble sugars, free amino acids, and leaf RWC; increased Chl levels; restricted the accumulation of toxic Na^+^, while enhancing other minerals uptakes, like K^+^ and Ca^2+^; improvement of yield-related traits	Khan et al. 2022b
Salinity	*Fragaria* × *ananassa*	40 mM NaCl, Not specified	Foliar application (1 and 2 mM)	Under salt stress, acetic acid application increased the accumulation of phytohormones, like ABA, CK, SA and GA; increased antioxidant enzyme activities, including SOD and CAT	Mirfattahi and Eshghi 2023
Alkaline salt	*Vitis vinifera*	100 mM NaHCO_3_, 7 days	Applied to the root zone (25 mM)	Enhanced endogenous level of acetic acid; increased the accumulation of SA; Upregulated the expression of *phenylalanine ammonialyases* (*PALs*)	Lv et al. 2021
Cadmium (100 mM)	*Solanum lycopersicum*	100 mg Kg^‒1^ of soil, 60 days	Roots treated with acetic acid-producing endophytic bacteria *Lysinibacillus fusiformis* Cr33	Enhanced rhizosphere Fe bioavailability; increased root acetic acid concentration, which subsequently activates JA signaling pathway and inhibits NO burst; restrict the accumulation of Cd	Zhu et al. 2021
Copper (0.3 and 3.0 mM)	*Lens culinaris*	0.3- and 3.0-mM CuSO_4_; 4 days	Added to the nutrient solution (10 mM)	Increased enzymatic antioxidant activities, like CAT and MDHAR, as well as the levels of GSH and GSSG; reduced the levels of MDA and electrolyte leakage; restricted the accumulation of toxic Cu, increased the levels of proline and leaf water content; increased Chl levels	Hossain et al. 2020
Arsenic (As)	*Pteris vittate*	Soils collected from As-contaminated area	Pretreatment to soil (10 and 20 mM)	Increased As accumulation in shoots and thereby recommended as a phytoremediation strategy	Wang et al. 2023
Cold and freezing stress	*Citrus aurantifolia*	0°C (cold stress) and − 6°C (freezing stress), 6 h (cold stress) and 30 h (freezing stress)	Pretreatment as foliar application (15 mM)	Increased enzymatic antioxidant activities, like POD, SOD and CAT under cold stress, while total phenolic acid content under freezing stress; reduced electrolyte leakage and MDA for both stresses; increased Fv/Fm and Fv/F_0_ ratio in both stresses	Sanie Khatam et al. (2023)

*Abbreviations: ABA* (abscisic acid), *ALDH2B7* (aldehyde dehydrogenase), *APX* (ascorbate peroxidase), *As* (arsenic), *AsA* (ascorbate), *Ca*^*2*+^ (calcium ion), *CAT* (catalase), *Cd* (cadmium), *Chl* (chlorophyll), *CK* (cytokinins), *Cu* (copper), *Fv* (variable fluorescence), *Fm* (maximal fluorescence), *GPX* (glutathione peroxidase), *GR* (glutathione reductase), *GSH* (reduced glutathione), *GSSG* (oxidized glutathione), *GST* (glutathione *S* transferase), HSPs (heat shock proteins), *IAA* (auxin), *JA* (jasmonic acid), *K*^+^ (potassium ion), *Mg*^*2*+^ (magnesium ion), *MDA* (malondialdehyde), *MDHAR* (mono-dehydroascorbate reductase), *MeJA* (methyl jasmonate), *Na*^+^ (sodium ion), *NCED2* (NINE-CIS-EPOXYCAROTENOID DIOXYGENASE 2), *NCED3* (NINE-CIS-EPOXYCAROTENOID DIOXYGENASE 3), and *NCED9* (NINE-CIS-EPOXYCAROTENOID DIOXYGENASE 9), *NO* (nitric oxide), *OST1* (OPEN STOMATA 1), *PDC1* (pyruvate decarboxylase), *PP2C* (protein phosphatase 2C), *RWC* (relative water content), *SA* (salicylic acid), *SOD* (superoxide dismutase), *TSPO* (outer membrane tryptophan-rich sensory protein

Similarly in rice (*Oryza sativa*), Ogawa et al. (2021) demonstrated that the treatment of plants with acetic acid elicits similar responses to drought stress by triggering JA signals (Table 1). Nonetheless, the induction of drought avoidance in rice through exogenously applied acetic acid does not occur through histone acetylation, as seen in Arabidopsis. Instead, acetic acid stimulates JA signaling and the accumulation of ABA in the roots, producing a pseudo-drought response characterized by decreased xylem flow from roots to shoots, reduced transpiration rate, and elevated alkalization of xylem sap (Table 1). The incorporation of acetic acid into root cells as acetate anions has been linked to the induction of membrane depolarization, a response similar to that triggered by herbivore attacks. This depolarization is further believed to initiate the biosynthesis of JA. However, further investigation is needed to fully comprehend the mechanism behind acetic acid-induced activation of the JA signaling pathway. The resilience of rice plants to drought stress can be attributed, at least in part, to the reduction in transpiration rate brought about by the interplay between the JA/ABA signaling mechanism in the shoot. The activation of the JA signaling pathway resulted in stomatal closure, principally orchestrated by ABA (Fig. 2). Despite these advancements, the precise signaling molecule responsible for transmitting the pseudo-drought response signal from the roots to the shoot remains elusive. Interestingly, acetic acid itself is not the source of the stress signal; rather, it subtly modulates some other unidentified factors that needs to be further studied and investigated.

It has been extensively documented that the external application of acetic acid not only significantly impacts the drought tolerance of crop plants, but also plays a critical role in mitigating drought stress in woody plants, such as cassava (Utsumi et al. 2019) (Table 1). External application of low levels of acetic acid significantly improved the drought tolerance of cassava plants by elevating the expression of ABA signaling genes (such as *OPEN STOMATA 1* and *protein phosphatase 2C*) and drought response/tolerance genes (such as *TSPO* and *heat shock proteins*). The increased expression of these genes led to a reduction in stomatal conductance and transpiration rate, ultimately leading to the plants maintaining a higher water status during drought stress (Utsumi et al. 2019). Additionally, foliar application of acetic acid to drought-stressed soybean plants at the V1 stage (fully developed first trifoliate) resulted in a significant decrease in stomatal conductance and transpiration rate, while simultaneously increasing the photosynthetic rate and water use efficiency (Rahman et al. 2020). Moreover, the soybean plants exposed to drought and treated with acetic acid displayed an enhanced osmotic adjustment, attributed to the increased level of both total soluble sugars and free amino acids (Table 1). Intriguingly, acetic acid can also act as an antitranspirant agent. Allen and Allen (2021) reported in their study on begonia plants that the low dose of acetic acid application protected this horticultural plant from over three weeks of drought stress by decreasing the rate of evaporation from the tray (Fig. 2). Additionally, the comprehensive economic analysis showed that utilizing a low dose of acetic acid resulted in a reduction of retail costs for begonia plants by 0.6% due to a 9-day extension in their lifespan. However, to ensure the maintenance of flower yield and avoid economic losses, investigation of the effective dosing of acetic acid as an antitranspirant agent is of utmost importance.

Li et al. (2021) revealed that the pretreatment of cotton plants (*Gossypium hirsutum*) with acetic acid via irrigation led to a substantial reduction in both stomatal conductance and transpiration rate. Additionally, the expression of genes involved in ABA and JA biosynthesis was dramatically enhanced, indicating a synergistic interaction between these hormones in reducing transpiration and stomatal conductance rate, thereby preserving more water for the survival of cotton during drought conditions (Fig. 2) (Table 1). Nevertheless, Sun et al. (2022) provided insight into the mechanism driving ABA and JA stimulation following acetic acid application, highlighting that drought stress triggered the release of SnRK2. This, in turn, activated the phosphorylation of AREB/ABF transcription factors, ultimately fostering the expression of ABA-responsive genes in apple plants (*Malus domestica*) under drought-stress conditions. Furthermore, the addition of acetic acid was found to induce the activation of the ABA-induced MAPK signaling pathway, resulting in elevated expression of *NCED* (MD05G0173700, MD05G0237600, and MD10G0164700), and heightened levels of ABA which further helped the plants to reduce water loss and increase drought tolerance. It is a well-established fact that the biosynthesis of JA originates from α-linolenic acid in the octadecanoid pathway that is facilitated by key enzymes like oxophytodienoic reductases (OPR), allene oxide cyclase (AOC), allene oxide synthases (AOS), and lipoxygenase (LOX) (Turner et al. 2002). A significant increase in the expression of genes responsible for the production of LOX, OPR, and AOS, and a consequent accumulation of JA in plants subjected to drought stress was augmented with acetic acid application. In contrast, a study by Kong et al. (2022) found that the application of acetic acid led to a decrease in ABA content in both roots and shoots of drought-stressed *Salix myrtillacea* plants while simultaneously elevating the levels of JA in both roots and leaves and  SA in leaves alone (Fig. 2). These findings suggest that acetic acid can regulate plant growth and physiological processes during drought by indirectly inducing the production of JA and SA.

In addition to enhancing the photosynthetic capacity of a variety of plants, acetic acid has also been demonstrated to mitigate oxidative stress by strengthening the antioxidant defense system of plants through the elevation of both enzymatic antioxidant activities and non-enzymatic antioxidant levels (Table 1). The application of acetic acid to soybean plants was reported to increase the activities of key antioxidant enzymes, superoxide dismutase (SOD), ascorbate peroxidase (APX), catalase (CAT), glutathione peroxidase (GPX), and glutathione *S*-transferase (GST), leading to a reduction in drought-induced oxidative stress (Rahman et al. 2020) (Fig. 2) (Table 1). Similar observation on acetic acid-mediated quenching of ROS due to reinforcement of antioxidant defense system has been reported in a variety of drought-stressed plants (Table 1) (Li et al. 2022; Kong et al. 2022; Sun et al. 2022).

 Drought exerted a profound impact on microbial activity, physiology, and habitat, which led to a substantial alteration in microbial community compositions (Omae and Tsuda 2022). Despite the wealth of research on the adaptive mechanisms mediated by acetic acid in plants, particularly in their aboveground parts under drought stress, the influence of acetic acid on soil microbial dynamics and its subsequent effect on plant performance remains a largely unexplored research area. A recent study by Kong et al. (2022) shed substantial insights into the effect of acetic acid on soil microbial dynamics and willow plant (*Salix myrtillacea*) performance under drought stress. This study reported that acetic acid irrigation (100 mL of 100 mM, 5 times total) dramatically boosted the abundance of *Azotobacter* and *Pseudomonas* in the soil, which was linked to elevated levels of critical soil metabolites such as glycyl-L-tyrosine, L-glutamine, and seryl-tryptophan. This, in turn, allowed *S. myrtillacea* plants to absorb more nitrogen, fortifying their resilience to drought conditions (Fig. 2). More fundamentally, it is well-recognized that glutamine plays a crucial role in nitrogen assimilation in plants, serving as a vital intermediate in the conversion of inorganic nitrogen into organic forms that are essential for the plant’s growth and development (Lee et al. 2023).

It is crucial to underscore that the central hub for plant–microbe interactions lies within the rhizosphere and root zone, where the attraction of microbes is regulated by the secretion of diversified root exudates (Stassen et al. 2020; Liu et al. 2020). For instance, the absence of coumarin biosynthesis, a phenylpropanoid metabolite produced by plants, can drive the growth of *Pseudomonas* (Voges et al. 2019). This finding was supported by the study conducted by Kong et al. (2022), which demonstrated that the reduction of phenylpropane metabolites, such as fraxetin and sinapyl aldehyde, resulted in a marked increase in the abundance of *Pseudomonas* in the soil under acetic acid irrigation and drought stress. In fact, the physical and chemical characteristics of the soil play a crucial role in shaping soil microbial communities, emphasizing the importance of exploring the full impact of acetic acid on both these properties and microbial dynamics in future research. Nonetheless, to verify the reliability and potential influence of acetic acid in enhancing drought tolerance under water-scarce conditions, well-designed field experiments should be performed in the presence and absence of acetic acid treatments.

### Acetic acid enhances salt stress tolerance in plants

In addition to mitigating the adverse effects of drought stress, acetic acid also plays a crucial role in mitigating the detrimental effects of salinity on plants. Rahman et al. (2019) reported  that foliar application of acetic acid protected mung bean plants from the detrimental effects of seawater-induced salt toxicity (Fig. 2). Acetic acid neutralized the damaging effects of salinity by modulating various physiological and biochemical processes, such as restricting the influx and build-up of toxic Na^+^, augmentating leaf succulence, and increasing photosynthetic rate, ion homeostasis, and osmoprotectant accumulation, as well as curbing salinity-induced oxidative stress by reinforcing antioxidant defense system (Table 1). Zhang et al. (2021) observed that salinity invoked a substantial increase in the endogenous acetic acid content in ryegrass (*Lolium perenne*), as manifested by a remarkable increase in the expression levels of *PDC1* (Pyruvate* decarboxylase 1*). They further confirmed the positive role of acetic acid in salinity tolerance with its exogenous application.  A significant increase in the K^+^ content and a decrease in the Na^+^ content was recorded in ryegrass leaves treated with acetic acid, leading to a higher K^+^/Na^+^ ratio compared to leaves treated with water (Fig. 2). Further investigation revealed that acetic acid treatment in ryegrass under salinity induced the expression levels of *HKT1;1* (*High-affinity K*^+^ *transporter 1*), a gene coding for a Na^+^ specific transporter or Na^+^/K^+^ co-transporter (Zhang et al. 2021). This observation suggests a correlation between the regulation of ion homeostasis and salt tolerance in perennial ryegrass by acetic acid. However, the molecular mechanisms underlying acetic acid-mediated suppression of the Na^+^ uptake and transport from roots to shoots remain unexplored and warrant further investigation. Khan et al. (2022b) presented ample evidence of the potency of foliar applications of acetic acid in the mitigation of salinity effects on wheat. Their study revealed a substantial enhancement in photosynthetic pigment contents and a surge in the rate of photosynthesis and transpiration along with increased acquisition of crucial nutrients, including Ca^2+^, K^+^, Mg^2+^, N, and P and a notable decrease in the influx of toxic ions like Na^+^ and Cl^─^ (Fig. 2). Additionally, the acetic acid-induced activities of enzymatic antioxidants managed to quench the salinity-induced oxidative burden indicated by the decreased levels of malondialdehyde, hydrogen peroxide, and electrolyte leakages in these plants (Fig. 2). The findings of Hossain et al. (2019) demonstrated the influential effect of external sodium-acetate application in resuscitating lentilplantsfrom salt-induced damage. This was achieved through a decline in shoot Na^+^ accumulation and a substantial increase in leaf ascorbate content, which reduced oxidative damage and membrane disruption (Hossain et al. 2019) (Table 1).

Acetic acid demonstrated a decisive impact in regulating phytohormone levels, thereby contributing significantly to the plant’s ability to overcome the damaging effects of salinity. Zhang et al. (2021) unveiled the effect of the acetic acid application under salt stress in triggering a substantial upsurge in the relative expression of *TAA1* (*tryptophan aminotransferase of Arabidopsis 1*), the gene responsible for IAA biosynthesis, and *ARF1* (*ADP-ribosylation factor 1*) and *ARF2* (*ADP-ribosylation factor 2*), two crucial IAA signaling pathways genes. Meanwhile, acetic acid application caused a decrease in the expression of *ZEP* (*zeaxanthin epoxidase*), a gene involved in ABA biosynthesis, and a significant reduction in the transcription levels of ABA signaling pathway genes *ABI3* (*abscisic acid insensitive 3*) and *ABI5* (*abscisic acid insensitive 5*) under both salt stress and normal conditions (Fig. 2). Furthermore, this study also reported that ryegrass plants treated with acetic acid substantially elevated the levels of JA, JA-Ile, and cis-OPDA (Oxylipin 12-oxo-phytodienoic acid, the precursor of JA). Lv et al. (2021) found that acetic acid significantly enhanced the tolerance of grapevines to alkali stress (NaHCO_3_) by elevating both the levels of acetic acid in vines and the biosynthesis of SA via the phenylalanine ammonia-lyase (PAL) pathway (Table 1). In line with these findings, Khan et al. (2022a) also observed elevated concentrations of phytohormones, including IAA and gibberellic acid (GA), coupled with a reduction in ABA content (Fig. 2). In contrast, Mirfattahi and Eshghi (2023) unveiled a significant increase in ABA levels following the application of acetic acid (2 mM), accompanied by elevated levels of cytokinin (CK), GA, and SA, indicating that the concentration of ABA is subject to fluctuations based on the type of stress encountered, where drought stress typically resulting in a rise in ABA accumulation, while salt stress leads to either increase or decrease. Nonetheless, there is a pressing need for further research to uncover the underlying molecular mechanism behind the interplay of acetic acid and phytohormones during salinity stress, as well as dose–response relationships and plant-specific responses.

In addition to the phytohormone modulation, acetic acid has been reported to profoundly orchestrate lipid metabolism (Hu et al. 2021). The findings of Hu et al. (2021) represent pivotal evidence exhibiting the potential of acetic acid to alleviate the damaging effects of salinity in turf grass (*Carex rigescens*) through the reprogramming of lipid metabolism (Fig. 2). The crucial factors that are associated with lipid metabolism under acetic acid treatment include (i) the substantial improvement in the accumulation of lipids such as phosphatidylcholine, phosphatidylethanolamine, phosphatidylglycerol, monogalactosyl diacylglycerol, galactosyl diacylglycerol, and sulfoquinovosyldiacylglycerol, which not only constitute a robust membrane lipid bilayer but also maintain membrane integrity against abiotic stress (Fig. 2), (ii) the significant enhancement of lipid signaling molecules and their precursors (phosphatidic acid, phosphatidylcholine, and phosphatidylinositol), which activate critical pathways such as CDP-choline pathway and phosphatidic acid, and defend the plants against salt stress (Fig. 2), and (iii) the dramatic reduction oflipid intermediates such as free fatty acids, reducing cellular damage and safeguarding the plant survival. It is imperative to delve deeper understanding of the mechanisms that drive the specific responses of lipids under acetic acid priming-conditions for enhancing salt tolerance. Despite the recent advances made toward understanding the mechanisms of acetic acid-induced salt tolerance in plants, there is a pressing need for comprehensive genomic, transcriptomic, proteomic, and metabolomic studies. Additionally, it is imperative to conduct extensive field studies to determine the optimal dose and application rates, and methods of acetic acid applications for various crops under different eco-physiological conditions.

### Acetic acid alleviates heavy metal toxicity in plants

In recent years, the potential of acetic acid to alleviate heavy metal toxicity in various crop plants has been identified (Zhu et al. 2021; Hossain et al. 2019, 2020, 2022). Zhu et al. (2021) demonstrated the involvement of acetic acid in root endophytic bacteria-mediated lower accumulation of Cd in tomato plants. Indeed, Cd toxicity often emerges from the interference of essential element uptake, especially iron (Fe), resulting in leaf chlorosis that resembles the symptoms observed in Fe deficiency. *Lysinibacillus fusiformis* generates a surplus of acetic acid, which dramatically enhanced the bioavailability of Fe in the rhizosphere and root cell walls, thus minimizing the toxicity of Cd (Zhu et al. 2021) (Fig. 2). It was also reported that the significant increase in acetic acid levels stimulated the JA signaling pathway that dramatically reduced root nitric oxide burst and alleviated the root Fe shortage caused by Cd stress. Intriguingly, they examined *L. fusiformis* inoculated plant responses under Cd stress in the presence of JA biosynthetic inhibitor sodium diethyldithiocarbamate and found that the ameliorative role of *L. fusiformis* on Cd toxicity was impeded, further confirming JA’s role in bolstering Cd tolerance. The overall synergistic impact of these events prevents Cd from entering root cells and thus effectively combats Cd toxicity in plants (Table 1). In another study, Hossain et al. (2022) revealed that acetic acid reduced Cd translocation from roots to shoots, strengthened the antioxidant defense system, and substantially diminished oxidative stress indicators in Cd-stressed lentil seedlings. Similarly, lentil seedlings, when pre-treated with 10 mM Na-acetate and subjected to two different concentrations of sodium arsenate (250 and 320 µM) for a duration of four days, showed increased tolerance to arsenate toxicity. This tolerance was evidenced by a decrease in arsenic accumulation in the roots and limited translocation to the shoots. Additionally, there was an enhancement in chlorophyll content and a reduction in oxidative stress, achieved through the reinforcement of the antioxidant defense system (Hossain et al. 2023). Lentil seedlings, on the other hand, when pretreated with Na-acetate followed by exposure to copper (Cu) stress, displayed reduced Cu accumulation in both roots and shoots, elevated proline levels, and strengthened antioxidant defense mechanism, thereby mitigating Cu-induced cellular oxidative damage, and elevating their resilience to Cu stress (Table 1) (Hossain et al. 2019). However, the elusive understanding of acetic acid-mediated heavy metal tolerance in plants requires a focused and in-depth investigation by incorporating comprehensive genomic, transcriptomic, proteomic, and metabolomic analyses under both lab and natural settings.

### Effect of acetic acid on cold stress responses in plants

Acetic acid also plays a crucial role in alleviating cold stress effects in plants. Recently, Sanie Khatam et al. (2023) unveiled that acetic acid also played a pivotal role in bolstering cold tolerance (0 °C) by fortifying enzymatic antioxidants, including POD, SOD and CAT, whereas in freezing tolerance (-6 °C), non-enzymatic antioxidant like phenol was found to play a crucial role. This study also reported the elevated levels of total soluble carbohydrates, photosynthetic pigments, and maximum quantum yield of photosystem II (Fv/Fm) in both cold and freezing stressed plants treated with acetic acid. Nevertheless, there is a need for further research to delve into the molecular mechanisms, elucidating the biochemical pathways involved, through which acetic acid could enhances cold tolerance in crop plants.

## Conclusions and future perspectives

Acetic acid has gained widespread recognition as a highly versatile substance, having been consistently utilized as a household vinegar, medicinal agent, and potent herbicide for weed control. A wealth of research also indicates that the external application of acetic acid plays a promising role to mitigate the adverse effects of abiotic stresses on plants. By functioning as a pivotal signaling molecule, acetic acid profoundly influences various physiological processes under abiotic stresses, contributing to enhanced photosynthesis rates, nutrient balance maintenance, fortification of antioxidant defense systems against oxidative stress, regulating phytohormonal levels, and accumulating protective metabolites to provide osmoprotection. It also  counteracts membrane disintegration and maintains essential lipid levels to ensure the stability and permeability of plant membranes. Additionally, the application of acetic acid has been observed to significantly enhance the abundance of soil microorganisms, particularly *Azotobacter* and *Pseudomonas*.

These positive effects imply the potential of acetic acid in plant resilience mechanisms. Thus, it is crucial to unravel the endogenous metabolism of acetic acid in plants, encompassing synthesis, degradation, and regulatory mechanisms under diverse stress conditions. Identifying cellular targets of acetic acid and examining its interactions with other signaling molecules like ethanol, melatonin, and phytohormones will enhance our understanding of complex regulatory networks in plant responses to abiotic stress. However, while positive effects of acetic acid are obvious, there is still a lack of research in validating the efficiency of acetic acid in mitigating abiotic stresses on crops, particularly in real field situations. Therefore, field trials are necessary to assess the practical and scalable application of acetic acid under diverse environmental conditions. Research efforts should also focus on optimizing application methods and determining appropriate quantities of acetic acid for broad-scale agricultural use. Moreover, understanding the interaction between acetic acid and plant microbiota is crucial. Investigating how acetic acid affects the abundance and diversity of soil microorganisms, particularly beneficial ones like *Azotobacter* and *Pseudomonas*, provides valuable insights into its overall impact on soil health and crop growth. Most importantly, the potential of acetic acid in boosting the shelf life of horticultural crops requires further investigation for potential industrial applications in horticulture. Overall, this chemical technology holds great promise for growers facing abiotic constraints in growing their crops, particularly in regions where transgenic cultivars may not be feasible or affordable.

## Data Availability

No data was used for the research described in the article.

## Abbreviations

ABA: Abscisic acid
ADH: Alcohol dehydrogenase
ALDH: Aldehyde dehydrogenase
AOS: Allene oxide synthases
APX: Ascorbate peroxidase
CAT: Catalase
CK: Cytokinin
EPA: Environmental Protection Agency
FIFRA: Federal Insecticide, Fungicide, and Rodenticide Act
GPX: Glutathione peroxidase
GST: Glutathione *S*-transferase
GA: Gibberellin
IAA: Indole 3 acetic acid
JA: Jasmonic acid
LDH: Lactate dehydrogenase
LOX: Lipoxygenases
NADH: Nicotinamide adenine dinucleotide
OPR: Oxophytodienoic reductases
PET: Polyethylene terephthalate
PAL: Phenylalanine ammonia-lyase
ROS: Reactive oxygen species
SA: Salicylic acid
SOD: Superoxide dismutase
WT: Wild type
